# Daikenchuto (TU‐100) alters murine hepatic and intestinal drug metabolizing enzymes in an in vivo dietary model: effects of gender and withdrawal

**DOI:** 10.1002/prp2.361

**Published:** 2017-10-03

**Authors:** Kentaro Nobutani, Jun Miyoshi, Mark W. Musch, Mitsue Nishiyama, Junko Watanabe, Atsushi Kaneko, Masahiro Yamamoto, Masaru Yoshida, Toru Kono, Hyunyoung Jeong, Eugene B. Chang

**Affiliations:** ^1^ Department of Medicine Knapp Center for Biomedical Center The University of Chicago Chicago Illinois; ^2^ Tsumura Research Laboratories Tsumura & Co. Ami Ibaraki Japan; ^3^ Division of Gastroenterology Department of Internal Medicine Kobe University Graduate School of Medicine Kobe Hyogo Japan; ^4^ Center for Clinical and Biomedical Research Sapporo Higashi Tokushukai Hospital Sapporo Hokkaido Japan; ^5^ Division of Gastroenterologic and General Surgery Department of Surgery Asahikawa Medical University Asahikawa Hokkaido Japan; ^6^ Departments of Pharmacy Practice and Biopharmaceutical Sciences College of Pharmacy University of Illinois Chicago Illinois

**Keywords:** Drug metabolizing enzymes, drug transporters, ginger, ginseng, sansho pepper

## Abstract

Herbal medicines and natural products used for maintenance of health or treatment of diseases have many biological effects, including altering the pharmacokinetics and metabolism of other medications. Daikenchuto (TU‐100), an aqueous extract of ginger, ginseng, and Japanese green pepper fruit, is a commonly prescribed Kampo (Japanese herbal medicine) for postoperative ileus or bloating. The effects of TU‐100 on drug metabolism have not been investigated. In this study, we analyzed the effect of TU‐100 on expression of key drug‐metabolizing enzymes (DMEs) and drug transporters (DTs) in murine liver and gastrointestinal tract using a dietary model. Liver, jejunum, and proximal colon were analyzed for phase I and II DMEs and DT mRNA expression by reverse transcription (RT) first by nonquantitative and followed by quantitative polymerase chain reaction (PCR) and protein expression. Liver, jejunum, and proximal colon expressed some identical but also unique DMEs and DTs. TU‐100 increased the greatest changes in cytochrome (Cyp) 2b10 and Cyp3a11 and Mdr1a. Basal and TU‐100 stimulated levels of DME and DT expression were gender‐dependent, dose‐dependent and reversible after cessation of TU‐100 supplementation, except for some changes in the intestine. Quantitative Western blot analysis of protein extracts confirmed the quantitative PCR results.

**Abbreviations**
Cyp: cytochrome P450
DME: drug‐metabolizing enzymes
DME: drug‐metabolizing enzymes
DT: drug transporters
Mdr1a: multidrug resistance protein 1a
RT: reverse transcription
Sult: Sulfotransferase
Ugt: UDP‐glucuronosyltransferase

## Introduction

A variety of herbal products are consumed worldwide, some prescribed and some taken over the counter. One safety concern regarding herbal medicines is their potential interaction with other coadministered drugs, resulting from changes of drug‐metabolizing enzymes (DMEs) and drug transporters (DTs) (Choi et al. 2011; Wanwimolruk and Prachayasittikul 2014 Cho and Yoon 2015). Many previous studies have shown the ability of herbal products, such as St. John's wort (*Hypericum Perforatum*) (Durr et al. 2000; Moore et al. 2000), ginkgo (*Ginko Biloba*) (Shinozuka et al. 2002; Gaudineau et al. 2004), garlic (*Allium sativum*) (Fisher et al. 2007), and turmeric (*Curcuma longa*) (Thapliyal and Maru 2001; Basu et al. 2004), to modify the expression and activity of phase I and phase II DMEs and DTs.

Liver and intestine are major sites for drug‐drug interactions, as both express phase I and phase II DMEs and DTs that can modulate pharmacokinetics of xenobiotic compounds and thus contributes to drug elimination (Choudhary et al. 2003, 2005; Alnouti and Klassen 2006; Buckley and Klaassen 2007; Van Herwaarden et al. 2007; Thelen and Dressman 2009; Giacomini et al. 2010; Renaud et al. 2011). Certain DMEs and DTs are expressed to a greater extent in the intestine than liver (Alnouti and Klassen 2006; Buckley and Klaassen 2007; Renaud et al. 2011; Fu et al. 2016). Of note, expression of DMEs and DTs show regional differences along the intestinal tract. For example, Cyp3a11 expression is greater in murine small intestine than in colon while Mdr1a expression increases from proximal to distal intestinal regions (Fu et al. 2016). Dietary and natural compounds such as grapefruit juice and St. John's wort have been shown to alter the metabolism of the drugs by affecting the expression of intestinal DMEs (Lown et al. 1997; Durr et al. 2000). Thus, it is important to investigate what contributions these compounds have on both intestinal and hepatic drug metabolism, taking into account how the impact of gender, dose, time course, and reversibility on their actions.

“Daikenchuto” (TU‐100) is a commonly prescribed Japanese herbal medicine that is used for the treatment of bowel symptoms and digestive diseases. TU‐100 is prescribed for bloating, constipation, and postoperative ileus by its ability to improve intestinal motility (Kono et al. 2015). TU‐100 is a mixture of extracts from a mixture of three different herbs, dried rhizome of *Zingiber officinale*, ripe fruit of *Zanthoxylum piperitum*, and root of *Panax ginseng* (Kono et al. 2015). The safety and efficacy of TU‐100 have been established and continues to be demonstrated in recent studies (Shibata et al. 1999; Manabe et al. 2010; Shimada et al. 2015; Katsuno et al. 2016). While previous studies reported potential drug‐drug interactions involving individual components of TU‐100 (i.e. ginger or ginseng) these studies were limited in that the combinatorial effects of multiple ingredients in TU‐100 were not assessed.

The present study employs a dietary model of TU‐100 in mice for a number of reasons. First, TU‐100 is consumed over long periods of time and effects may take months and continued consumption is required for maintained effects. A previous study on intestinal microbiome effects of TU‐100 demonstrated that changes were observed only after many weeks of ingestion (Hasebe et al. 2016). Second, the intestinal microbiome changes may be important for alterations of DMEs and DTs as intestinal bacteria have been determined to modulate their expression (Einarsson et al. 1973; Nugon‐Baudon et al. 1998; Hooper et al. 2001). Third, with dietary introduction in an in vivo model, expression of DMEs and DTs is regulated by all factors that naturally modulate liver and hepatic DME and DT expression. Ginseng, one component of TU‐100 has many components metabolized by intestinal bacteria (Qi et al. 2011). These metabolites may have different effects on DME and DT inhibition or induction. Thus, in vitro studies using cultured cells cannot recapitulate the multifactorial regulation of expression. Importantly, DMEs and DT are regulated not only through bacterial regulation, but also direct effects of compounds. These effects may occur by inhibition or induction of expression. The present study focuses on the regulation of mRNA and protein expression. Gene induction of DME and DT is regulated by many factors, including bacterial regulation (Einarsson et al. 1973; Nugon‐Baudon et al. 1998; Hooper et al. 2001) and nutritional factors (Jacobs and Lewis 2002). The identity of many of the transcription factors involved has been studied and includes the CXR, PXR, NF‐kB, and the aryl hydrocarbon receptor (Honkakoski and Negishi 2000; Gibson et al. 2002; Hyrcay and Bandiera 2009; Zordoky and El‐Kadi 2009; Tolson and Wang 2010; Wang et al. 2011);

Studies of DME and DT in the mouse must be regarded as effects in this species as the regulation of DMEs and DTs is different in human (Nelson et al. 2004; Martignoni et al. 2006; Wang et al. 2011). Substrates for mouse homologs have been correlated with human DMEs, particularly cytochrome P450 isoforms and the substrate defined families within the cytochromes P450s which may be different in the two species and their regulation may be different (Nelson et al. 2004; Martignoni et al. 2006; Wang et al. 2011). Studies of DME and DT in the mouse cannot be stated to occur for human homologs defined on substrate or gene identity, however, studies of DMEs and DT that have been performed in mice, rats, and rabbits have provided important information on regulatory events that occur in humans.

In this study, we analyzed the dose‐ and time‐dependent effects of TU‐100 on the mRNA and protein expression of major DMEs and DTs in different regions of the intestine and in the liver of both males and female mice. We also investigated the reversibility of these changes upon discontinuation of TU‐100 in the diet.

## Materials and Methods

### Mice

All animal experiments were approved by the University of Chicago Institutional Animal Care and Use Committee (IACUC protocol 71084). Forty‐eight male and 48 female C57Bl6/J mice from 5 to 10 weeks of age were bred in house. Littermates were then equally divided among the study groups. All mice were fed standard mouse chow, but then switched to a defined AIN‐76A diet (described below) 1 week before the beginning of the study. Mice were euthanized by carbon dioxide inhalation followed by cervical dislocation as approved by IACUC.

### Diet and drug

AIN‐76A is a defined diet purchased from Harlan Teklad/Envigo (Madison, WI; CA.170481), where all the components are known and standardized for consistency. “Daikenchuto” (TU‐100) was obtained as powder from Tsumura & Co. (Ami, Ibaraki, Japan). TU‐100 was included in AIN‐76A at 15 g TU‐100/kg diet (1.5% wt/wt) (Harlan Teklad; TD.110333). This dosage of TU‐100 was determined previously to obtain similar blood concentrations of major TU‐100 ingredients in mice to the human data (Kaneko et al. 2013; Kono et al. 2013; Ueno et al. 2014; Watanabe et al. 2015; Hasebe et al. 2016). For the present studies, doses of 0.75% and 3.0% wt/wt TU‐100 in the diet were also used to assess its dose‐dependency.

### Animal experimental procedures

**Figure 1:**
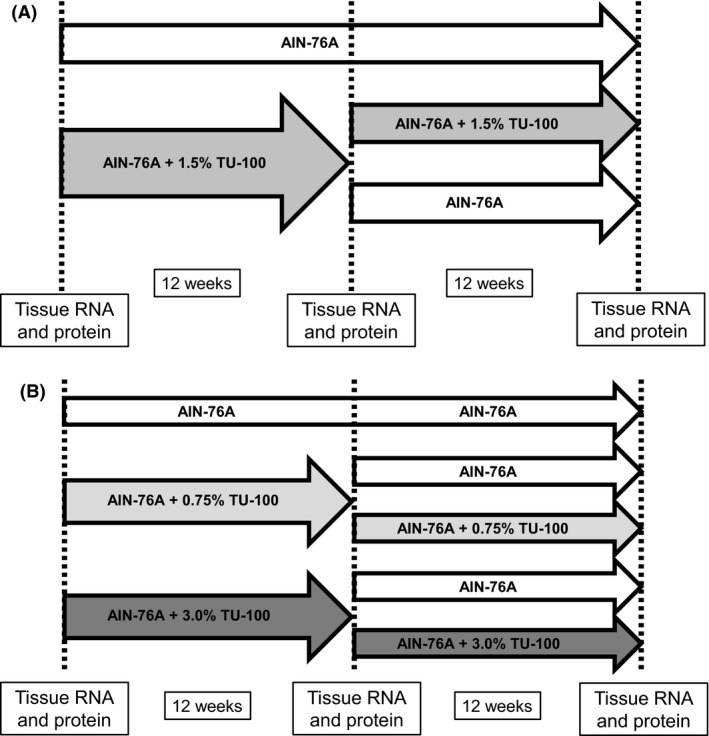
Experimental procedure: timelines of feeding protocols. (A) Male and female C57BL6/J mice were fed AIN‐76A, a defined, formulated diet, with or without 1.5% TU‐100 for 24 weeks. After 12 weeks, half of the mice fed with AIN‐76A with 1.5% TU‐100 were switched back to AIN‐76A without 1.5% TU‐100, and the remaining mice continued the same diet for the duration of the study. Tissue RNA and protein samples were collected at 12 and 24 weeks from 8 to 16 mice in each diet group. (B) Male C57BL6/J mice were fed with AIN‐76A without TU‐100 or AIN‐76A supplemented with 0.75% or 3.0% TU‐100 for 24 weeks. After 12 weeks, half of the mice were switched back to AIN‐76A without TU‐100, and the remaining mice continued the TU‐100 supplemented diet for a whole period. Tissue RNA and protein samples were collected at 12 and 24 weeks from 5 mice in each diet group.

Male and female mice were fed the AIN‐76A defined diet with or without 1.5% TU‐100 for 12 weeks. After 12 weeks, half of the mice were switched to the AIN‐76A diet without TU‐100, and the other half continued the same AIN‐76A diet with TU‐100 for another 12 weeks (Fig. 1A). Tissue samples from the liver, jejunum, and proximal colon were collected at 0, 12 and 24 weeks of the experiment from 8 to 16 mice in each diet group (Fig. 1A). To analyze the effect of different doses of TU‐100, 5 male mice in each diet group (AIN76A with no TU‐100, 0.75% TU‐100, or 3.0% TU‐100) were sacrificed and analyzed at 0, 12, and 24 weeks (Fig. 1B). A section of liver was immersed in TRIZOL reagent (Thermo Fisher/Invitrogen, Carlsbad, CA) then homogenized. The mucosal layer of the jejunum and proximal colon was scraped off from the tissue using glass slides, and immersed in TRIZOL reagent, then homogenized. The tissue samples were stored at −80°C until the RNA extraction. For protein extraction, samples of liver or intestinal scrapings were place into lysis buffer (10 mmol/L Tris pH 7.4, 1 mmol/L MgSO_4_ with 1 mmol/L PMSF and Roche Complete Protease Inhibitor). After homogenization using a teflon pestle, an aliquot was removed for protein determination by the bicinchoninic acid procedure and remainder was solubilized with 3X Laemmli SDS‐PAGE stop solution, heated and frozen for Western blotting analysis.

### Reverse transcription polymerase chain reaction (RT‐PCR)

RNA was extracted from the tissue homogenates in TRIZOL reagent following the manufacturer's instruction. cDNA was generated using Transcriptor First Strand cDNA Synthesis Kit (Roche, Indianapolis, IN) and then used first for nonquantitative PCR using Takara ExTaq (Fisher Scientific, Itasca, IL) using an annealing temperature of 56°C for all reactions. Thirty five cycles were used for all reactions Specific targets were selected for each tissue and quantitative PCR was performed using iTaq Universal SYBR Green Supermix (Bio Rad, Richmond, CA), and a Light Cycler 480 (Roche) according to manufacturer's protocol for SYBR Green I which uses a 60°C annealing temperature. Data were normalized to the amount of glyceraldehyde phosphate dehydrogenase (Gapdh) as an endogenous control.

For analysis of mRNA targets, all samples from one tissue for comparison were run in one PCR plate from a single master mix of reagents. A standard curve was generated for each target as well as Gapdh. This standard was diluted and used to generate a standard curve of Ct values of this calibrator sample. The expression level of each sample for that target was calculated by this standard calibrator curve. Each sample constitutive control, Gapdh, was calculated similarly. This analysis was used to account for variability in initial concentration and quality of total RNA and conversion efficiency in each reverse transcription sample. Values are presented as relative expression level (normalized to Gapdh of each sample).

### Oligonucleotide primers

Primers were designed based on previous reports or Primer Bank (https://pga.mgh.harvard.edu/primerbank) (Choudhary et al. 2003; Wang and Seed 2003; Spandidos et al. 2008, 2010; Xu et al. 2012) (Table S1).

### Western blotting

Samples were separated on 10% SDS‐PAGE and transferred to PVDF membrane by standard protocols. Antibodies used in this study included anti‐CYP2B10 (EMD Millipore AB9916, Temecula, CA), anti‐CYP3A11 (EMD Millipore MAB10041, Temecula, CA), anti‐beta actin (Cell Signaling #4967, Danvers, MA), anti‐p‐glycoprotein (Abcam ab170904, Cambridge, MA). Blots were developed using an enhanced chemiluminescence system (Western Bright, Advansta, Menlo Park, CA).

### Compliance with design and statistical analysis requirements

At least five mice were used for all experimental groups. Groups included mice from at least two different breeding pairs and were within one week of age for both male and female mice. Samples were run in duplicate and were averaged. Samples from the same tissue from all mice were analyzed on the same PCR plate to permit comparison. The fold changes of the gene expression levels of the TU‐100 fed mice were calculated by comparison to those of the control mice fed with AIN‐76A without 1.5% TU‐100. All results are expressed as means ± SEM. Outliers were identified and eliminated using Grubb's test using GraphPad Prism (San Diego, CA). Statistical significance was determined using unpaired Student's t‐test for comparison between two groups, and nonrepeated measures ANOVA, followed by the Student–Newman–Keuls test for comparison among more than two groups. A *P* < 0.05 was considered statistically significant.

## Results

**Figure 2:**
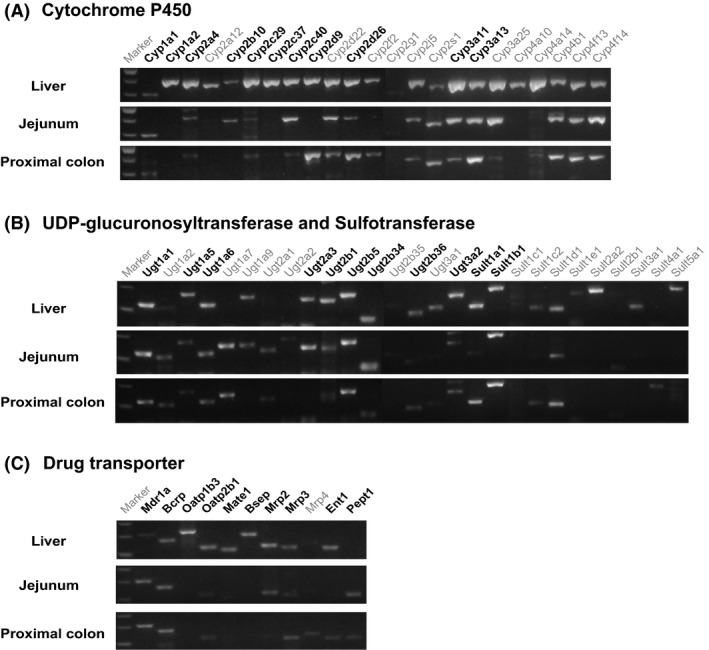
Tissue distributions of DMEs and DTs. mRNA expression levels of Cyp, Ugt, Sultt, and DT in the liver, jejunum, and proximal colon of female mice were analyzed by RT‐PCR and gel electrophoresis. The genes indicated by bold letters were subjected to the further analysis by real‐time quantitative RT‐PCR. (A) The expression levels of the Cyps. (B) The expression levels of the Ugts and Sults. (C) The expression levels of DTs. The sizes of the marker bands from the top are: (A) 500 bp, 400 bp, and 300 bp; (B) 300 bp, 200 bp, and 100 bp; (C) 300 bp, 200 bp, and 100 bp respectively.

To determine the tissue expression of DMEs and DTs, transcript abundance was examined by nonquantitative RT‐PCR followed by gel electrophoresis (Fig. 2). Liver, jejunum, and proximal colon samples from female mice at week 0 were analyzed. Gene expression patterns were consistent with previous reports, when available (Renaud et al. 2011; Fu et al. 2016). Liver expressed the largest number of DMEs and DTs, however, some were more abundant in the jejunum or proximal colon (Fig. 2). Similar results were obtained in male mice. Notably p‐glycoprotein (Mdr1a) was expressed at a greater level in jejunum and proximal colon (Fig. 2). The transporter PepT1 was not expressed in liver and only both sections of the intestine (Fig. 2). Liver expressed nearly every cytochrome tested and at greater levels than intestine. The only cytochromes expressed as slightly greater levels in intestine were Cyp1a1 and Cyp3a13. For phase II enzymes, intestine expressed more Ugt1a2, and liver expressed a greater number and higher levels of most Ugt and Sult enzymes. Target genes from the above were selected for real time quantitative PCR analysis by the following criteria: (1) The genes are expressed as bands detected in the nonquantitative PCR data (Fig. 2); (2) Activity of these mouse DMEs and DTs genes have substrate specificity similar to human DMEs and DTs considered important for drug metabolism; (3) The genes are members of subfamilies of DME and different types of DT.

**Figure 3:**
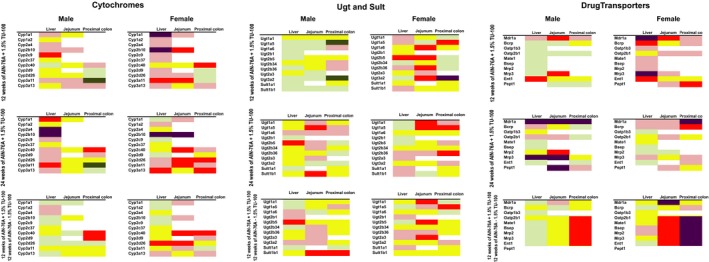
Heat map presentation of TU‐100 induced DMEs and DTs changes. Male and female C57BL6/J mice were fed with AIN‐76A defined diet with or without 1.5% TU‐100 for 12 weeks (top panels) or 24 weeks (middle panels). After 12 weeks, half of the mice fed with AIN‐76A with 1.5% TU‐100 were switched to AIN‐76A without 1.5% TU‐100, and the remaining mice continued the original TU‐100 supplemented diet for the duration of the study (bottom panels). mRNA expression levels of the cyp, ugt, sults, and drug transporters were analyzed by RT‐PCR and calculated as fold changes as described in Methods. For presentation, a fold change in 1.0 indicates no change (represented as yellow). Decreases are represented in green, dark green for changes 0.25 to 0.5 and light green smaller decreased fold changes from 0.50 to 1.0. Light red represents increased fold changes from 1.0 to 1.5, dark red increased fold change from 1.5 to 2.0 and purple the greatest fold changes, over 2.0. Values (means ± standard deviation) are presented in a supplemental file.

**Figure 4:**
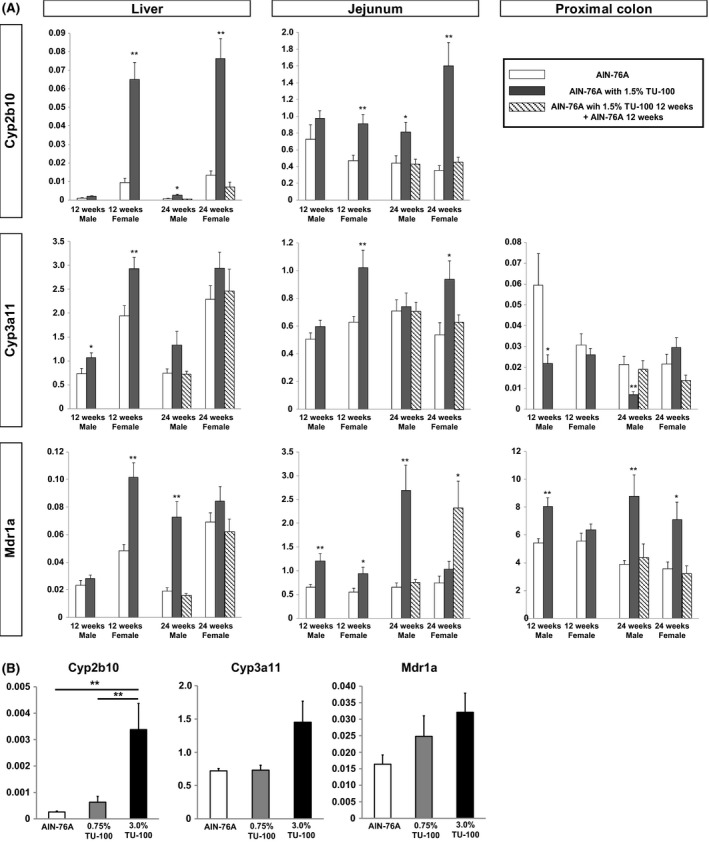
Expression levels of Cyp2b10, Cyp3a11, and Mdr1a in mice fed TU‐100 supplemented diet. (A) Male and female C57BL6/J mice were fed with AIN‐76A defined diet with or without 1.5% TU‐100 for 24 weeks. After 12 weeks, half of the mice fed with AIN‐76A with 1.5% TU‐100 were switched to AIN‐76A without 1.5% TU‐100, and the remaining mice continued the original TU‐100 supplemented diet for the duration of the study. mRNA expression levels of the Cyp, Ugt, Sults, were analyzed by RT‐PCR. Data shown are relative mRNA expression levels of Cyp2b10, Cyp3a11, and Mdr1a normalized to Gapdh (*n* > 8). Statistical significance was determined using unpaired Student's t‐test comparing to the mice fed with AIN‐76A without 1.5% TU‐100 (*P < 0.05, **P < 0.01). (B) Male C57BL6/J mice were fed AIN‐76A defined diet without TU‐100 or AIN‐76A including 0.75% or 3.0% TU‐100 for 24 weeks. mRNA expression levels of Cyp2b10, Cyp3a11, and Mdr1aa in the liver were analyzed by RT‐PCR. Data shown are relative mRNA expression levels of Cy;2b10, Cyp3a11, and Mdr1a were normalized to Gapdh (*n* = 5). The y axis for all samples is relative expression level (normalized to Gapdh of each sample first). Statistical significance was determined using non‐repeated measures ANOVA, followed by the Student–Newman–Keuls test (**P < 0.01). Y axis: relative gene expression levels to Gapdh.

Fold changes in the gene expression of the 1.5% TU‐100 fed mice at 24 weeks as well as those fed diet with 1.5% TU‐100 for 12 weeks followed by 12 weeks without TU‐100 were analyzed with respect to that in control mice fed AIN‐76A without TU‐100 at 12 weeks (Figs. 3 and 4). Fold changes are presented as a heat map representation in Fig. 3, values are presented in the supplemental file (Table S2). Unchanged values, a fold change in 1.0, are represented by yellow. Green was selected for decreased and red and purple for increased expression. Darkest green indicates fold changes were between 0.25 and 0.5, light green between 0.5 and 1.0. Light red indicates increased fold changes between 1,0 and 1.5, dark red fold changes between 1.5 and 2.0 and purple was used to indicate greatest changes, over 2.0. Twelve or 24 weeks dietary supplementation with 1.5% TU‐100 changed expression of a variety of DMEs and DTs both in male and female mice, although the fold change varied depending on tissue, gender, and duration of treatment (upper and middle rows of Fig. 3 and as labelled in Fig. 4). Most notable increases were in Cyp2b10 and 3a11 (Figs. 3 and presented as bars in Fig. 4A) and the transporters Mdr1a, Mrp2, Mrp3, and PepT1 (Fig. 3 and as bars in Fig. 4A). TU‐100 induction of some targets such as Mdr1a and Mrp2 increased between 12 and 24 weeks of dietary TU‐100 (Fig. 3). From Table 1 of File S2, some changes occurred more rapidly in female mice (Cyp2b10) with induction in males increasing significantly only after 24 weeks of dietary TU‐110 (Cyp 2b10 and 2a4). Many of the TU‐100 ‐induced DME and DT changes were reversed after 12 weeks of discontinuation of TU‐100 supplementation (bottom panels of Fig. 3). Most of the DME and DT changes reversed 12 weeks after termination and withdrawal of dietary TU‐100, the exception being jejunal Mdr1a and PepT1 where increased mRNA expression persisted. All DMEs induced by dietary TU‐100 were nearly completely reversed. TU‐100 induced changes were dependent on dietary amount. Male mice were fed diets of no TU‐100, 0.75% and 3% for 24 weeks. Samples were analyzed only for Cyp2b10, Cyp3a11, and Mdr1a (Fig. 4B). Mdr1a was the most responsive target and increased with 0.75% TU‐100 and 3% TU‐100 increased expression of both cytochromes.

**Figure 5:**
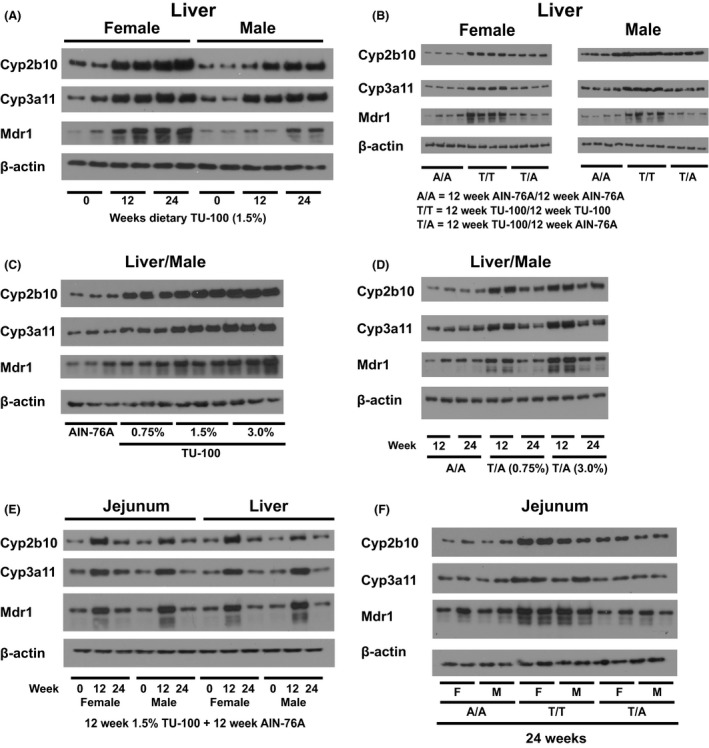
TU‐100 alters Cyp2b10, Cyp3a11, and Mdr1a protein expression in liver and jejunum. Protein samples were analyzed by 10% SDS‐PAGE and Western blots using specific antibodies. Beta actin was used as a housekeeping gene. (A) Liver tissue from female and male mice fed 1.5% TU‐100 containing AIN‐76A diet for 12 or 24 weeks. (B) Liver tissue from female and male mice fed AIN‐76A diet without TU‐100 for 24 weeks (A/A); 1.5% TU‐100 supplementation for 24 weeks (T/T); 1.5% TU‐100 supplementation for 12 weeks followed by AIN‐76A without TU‐100 for 12 weeks (T/A). (C) Liver tissue from male mice fed AIN‐76A diet without TU‐100, or 0.75, 1.5, or 3.0% TU‐100‐supplemented AIN‐76A diet for 12 weeks. (D) Liver, male mice fed AIN‐76A diet without TU‐100 for 12 or 24 weeks (A/A); 0.75% or 3.0% TU‐100 containing AIN‐76A diet for 12 weeks followed by the diet without TU‐100 for 12 weeks (T/A). (E) Jejunum and liver tissues from females and males fed 1.5% TU‐100 supplemented AIN‐76 diet for 12 weeks followed by the diet without TU‐100 for 12 weeks. (F) Jejunal tissue from female (F) and male (M) mice harvested at 24 weeks of AIN76A diet without TU‐100 (A/A), fed diet with TU‐100 for full 24 weeks (T/T), or fed diet with TU‐100 for 12 weeks followed by 12 weeks without TU‐100 (T/A) were analyzed.

To determine whether increases in mRNA changes were observed at levels of protein expression, liver and jejunal samples were analyzed by Western blotting, using beta actin as a housekeeping protein. To allow comparison, basal and induced protein expression in liver, samples of both genders were analyzed on the same blots using samples acquired at 12 and 24 weeks after dietary 1.5% TU‐100 (Fig. 5A). Increases in Cyp2b10, Cyp3a11, and Mdr1a were greater and more rapid in samples from female mice. To determine the reversibility of these effects, samples were analyzed at 24 weeks from mice of both genders fed AIN‐76A diet for 24 weeks (A/A), 1.5% TU‐100 containing diet for 24 weeks (T/T), or mice fed 1.5% TU‐100 containing diet for 12 weeks followed by the diet without TU‐100 for 12 weeks (T/A) (Fig. 5B). The increases in Cyp2b10, Cyp3a11, and Mdr1a by TU‐100 were reversible, as exemplified by the group of 4 to the far right in each blot. Induction occurred in a dose‐dependent manner shown by samples from male mice fed diets with either 0.75 or 3.0% dietary TU‐100 analyzed at 12 weeks of induction (Fig. 5). Reversibility of DME and DT induction in the liver at different doses was also observed in male mice fed the AIN‐76A diet with 0.75 or 3.0% of TU‐100 for 12 weeks followed a 12 weeks period on the same diet without TU‐100 (T/A) compared to their counterparts that were fed the AIN‐76A diet for the entire 24 weeks (A/A) (Fig. 5D). Therefore, jejunal samples from male and female T/A mice were analyzed further by Western blot at 0 and 12 weeks during which they were fed 1.5% TU‐100, and 12 weeks after cessation of TU‐100 (Fig. 4E). Liver samples were analyzed on the same blots to permit comparison (Fig. 5E). TU‐100 induced the cytochromes and Mdr1a in jejunum similar in a similar fashion to previous results in the liver in both male and female mice (Fig. 5E). Samples were analyzed from female (F) and male (M) mice maintained on AIN76A diet without TU‐100 for 24 weeks (A/A), those that had been switched to diet with TU‐100 for 24 weeks (T/T), and mice fed TU‐100 containing diet for 12 weeks and switched back to diet without TU‐100 for 12 weeks (T/A). These samples confirmed the induction and reversibility of Cyb2b10, Cyp3a11, and Mdr1a in jejunum (Fig. 5F).

## Discussion

The present study shows that dietary supplementation with the natural product TU‐100 modifies major DMEs and DTs of the murine liver and various regions of the intestine. Two DMEs, (Cyp2b10 and Cyp3a11) and the drug transporter (Mdr1a) were particularly affected in a manner that was both gender‐ and dose‐dependent. In addition, many, but not all of the induced transcript responses were reversible. We also found corresponding changes in protein expression to the observed changes in mRNA transcripts. These observations are noteworthy considering the wide usage of TU‐100 components in Japan and increasing interest in health‐promoting natural products in western countries. Furthermore, these studies were performed under carefully controlled conditions where the effects of gender, dose, duration of treatment, and reversibility of effects on DMEs and DTs were investigated.

Changes in DMEs and DTS in the present studies in mice may not be assumed to occur, even for gene homologs, in other species, including humans. Gene homologs have been identified based on sequence and enzyme homologs have also been identified based on substrates metabolized. Even for mouse and human homologous cytochromes that metabolize the same substrates, regulation of gene expression can be different. The present studies allow investigation of parameters not readily studied in humans as in vivo experimentation is required. In vitro studies of human liver or intestinal cells are valuable for DME and DT inhibition and induction studies, but do not recapitulate the role of intestinal bacteria to regulate DME and DT expression through bacterial metabolite generation, nor do in vitro studies recapitulate the role of intestinal bacteria play in drug and xenobiotic metabolism.

The present studies demonstrate changes in induction by TU‐100 but drug metabolism may also be regulated at the level of enzyme inhibition. DME inhibition may result in decreased metabolism of coadministered drugs. Of the three components of TU‐100, ginseng has been studied for inhibition of activity of human liver cytochromes (Liu et al. 2006). Naturally occurring ginsenosides had no effect to inhibit human CYP3A4, CYP2D6, CYP2C9, CYP2A6, nor CYP1A2. However, many ginenosides are metabolized by intestinal bacteria. Compound K, a ginsenoside Rb1 metabolite as well as two other ginsenoside metabolites, protopanaxadial and protopanaxatriol, exhibited moderate inhibition of CYP2C9 (Liu et al. 2006). Studies of TU‐100 (at extract concentrations up to 2000 *μ*g/mL) using rat, dog, and human liver microsomes cytochrome activity demonstrated no cytochrome inhibition (unpublished data). A bidirectional assay using Caco2 cultured human intestinal enterocytes, TU‐100 extract (up to 1671 *μ*g/nL) demonstrated some inhibition of p‐glycoprotein (Mdr1) activity (unpublished observations).

The level of expression of key DMEs and DTs varies among different organ systems. Cyp2b10 has been reported to be highly expressed in the lung, followed by the proximal small intestine with lower expression in the liver (Renaud et al. 2011). Many DMEs and DTs are expressed in the liver, probably accounting for why it historically has received the greatest attention. However, our study as well as others show that the gastrointestinal tract should be included in studies of xenobiotic metabolism (Van Herwaarden et al. 2007; Thelen and Dressman 2009).

The molecular mechanisms underlying altered expression of DMEs and DTs by TU‐100 could be potentially multifactorial. One possibility is that components of TU‐100 directly affect gene transcription by modulating the activities of nuclear receptors. Pregnane X receptor (PXR) and constitutive androstane receptor (CAR) are ligand‐activated nuclear receptors that are known to upregulate expression in both mouse and human liver. Currently available data, however, indicate that components of TU‐100 (i.e. ginseng) are not agonists of PXR (Zhang et al. 2015) although it remains to be determined whether other compounds of TU‐100 are capable of modulating PXR or CAR. Another possibility is the role of differential gut microbiome in altered expression of DMEs and DTs (Kuno et al. 2015). TU‐100 is known to alter the intestinal microbiome (Hasebe et al. 2016) and accumulating evidence indicate that gut microbiome plays a key role in regulating hepatic and intestinal DMEs and DTs (Einarsson et al. 1973; Overvik et al. 1990; Nugon‐Baudon et al. 1998; Hooper et al. 2001; Bjorkholm et al. 2009; Meinl et al. 2009; Toda et al. 2009; Saad et al. 2012; Selwyn et al. 2015). For example, Cyp activity measured by the microsomal metabolism of steroids was shown to be lower in the liver of germ‐free rats as compared with conventionally raised (Einarsson et al. 1973). Also, colonization of germ‐free mice with Bacteroides thetaiotamicron, a prominent component of the normal mouse gut microbiome, was shown to modulate intestinal Cyp and Mdr1a expression (Hooper et al. 2001). Additionally, germ‐free rats fed a diet supplemented with myrosinase‐free rapeseed exhibit different levels of expression of liver Cyp expression, demonstrating the complexity of action of dietary supplements that may involve direct and microbe‐dependent actions (Nugon‐Baudon et al. 1998). Both direct actions of TU‐100 and indirect actions via modulating the intestinal microbiome are likely involved and warrant further investigation.

In addition, differences in gender must be factored in when examining natural products like TU‐100 in affecting xenobiotic metabolism (Kato and Yamamoe 1992; Franconi et al. 2007). Clinically, age and gender are strong considerations when placing patients on medications. This is further complicated when dietary supplements such as TU‐100 are used, where their actions on drug metabolism are also influenced by age, dose, and gender. The time course and extent of reversibility upon cessation of TU‐100 is another clinically important consideration. Our study shows that most of the changes in DMEs and DTs are reversible in the mouse model, but some changes persist for months after cessation of TU‐100, especially in the intestine. This is similar to the sucralose‐based artificial sweetener (Splenda), a chlorinated sucrose which is not metabolized by small intestinal sucrase. In this study, altered composition of the gut microbiome and intestinal expression of certain Cyp and Mdr1a persisted for up to 12 weeks following cessation of the sweetener in rats (Abou‐Donia et al. 2008). From these observations, we speculate that the effects of TU‐100 on host DMEs and DTs are in part dependent on changes in the gut microbiome, and that the lasting effects upon its cessation are due to persistent changes in gut microbial communities.

In conclusion, dietary TU‐100 modulates both liver and intestinal expression of murine DMEs and DTs. The effect is gender and dose‐dependent and reversible in most cases. These conditions might be important for studies of drug interaction in humans.

## Disclosure

None declared.

## Supporting information


**Table S1.** Effect of TU‐100 on drug metabolizing enzymes/cytochromes Phase 1.

**Table S2.** Effect of TU‐100 on Drug Metabolizing Enzymes/Phase II.
**Table S3.** Effect of TU‐100 on drug transporters**.**
