# Supplementary material for: Daikenchuto (TU‐100) alters murine hepatic and intestinal drug metabolizing enzymes in an in vivo dietary model: effects of gender and withdrawal
Source: Pharmacol Res Perspect. 2017 Oct 3;5(5):e00361. doi: 10.1002/prp2.361 (PMC5625165; doi:10.1002/prp2.361)
Supplement: Supplementary file 1 — Table S1. Effect of TU‐100 on drug metabolizing enzymes/cytochromes Phase 1. [file PRP2-5-e00361-s001.rtf]

Supplemental Table . Primer sequences for reverse transcription PCR

Name	Sequences (Forward / Reverse)	Product Size (base pairs)	References	
Gapdh	AGGTCGGTGTGAACGGATTTG / TGTAGACCATGTAGTTGAGGTCA	123		
Cyp1a1	GGCCAGACCTCTACAGCTTC / GCCAAAGCATATGGCACAG	296	D. Choudhary et. al. (2003)	
Cyp1a2	GACGTCAGCATCCTCTTGCT / GGCACTTGTGATGTCTTGGA	400	D. Choudhary et. al. (2003)	
Cyp2a4	CTAAGGAGCTTCTCCATCGC / GCGCTGATTGTGTTCCACT	393	D. Choudhary et. al. (2003)	
Cyp2a12	TTGATCAAGATGTTGCAGGG / TTGCATGTGGATGAGAAAGG	363	D. Choudhary et. al. (2003)	
Cyp2b10a	GCCCAATGTTTAGTGGAGGA / GACTTCTCCTTCTCCATGCG	383	D. Choudhary et. al. (2003)	
Cyp2b10b	AAAGTCCCGTGGCAACTTCC / TTGGCTCAACGACAGCAACT	248	PrimerBank ID : 1684709a1	
Cyp2c29	AGGAAAACCAAAGGCTCACC / TGCTTGCCAGATTTTCAAGT	400	D. Choudhary et. al. (2003)	
Cyp2c37	GTTGCCTTGTGGAGGAACTT / AATGGTTGATTGCCGTCTTC	391	D. Choudhary et. al. (2003)	
Cyp2c40	AAAACAAATGGCTCACCCTG / TTGCCAGGTGTTCAATGGTA	394	D. Choudhary et. al. (2003)	
Cyp2d9	GAGCAGAGGCGATTCTCTGT / CCCAGGTGGTCCTATTCTCA	400	D. Choudhary et. al. (2003)	
Cyp2d22	GGCGCTTCTCTGTGTCTACC / GTCCCAGGTCGTCTTGTGTT	395	D. Choudhary et. al. (2003)	
Cyp2d26	AAAGGTGTGATCCTTGCACC / GAATGAATTCAGCTTGGGGA	393	D. Choudhary et. al. (2003)	
Cyp2f2	TGGGAAAAAGAAGCATCGAG / GAAGCAGTCGATGAAGTCCC	395	D. Choudhary et. al. (2003)	
Cyp2g1	GTGGACCAGGCAGATGACTT / ATAGTCGAAGCGTTTTCCGA	306	D. Choudhary et. al. (2003)	
Cyp2j5	ATGGCACTGAGGAACTTTGG / CTCTTGGCTCATCTGGGTTC	400	D. Choudhary et. al. (2003)	
Cyp2s1	GAGAAGGCGAGGAGCTGAT / CAGGACCTGAGGTTTGGAAG	366	D. Choudhary et. al. (2003)	
Cyp3a11	ATAGAGCTTTGCTGTCCCCC / CGGCTTTCCTTCATTCTGTC	394	D. Choudhary et. al. (2003)	
Cyp3a13	CCCTGCTGTCTCCAACCTT / TGCGATTCTCTTTCATTCGTT	390	D. Choudhary et. al. (2003)	
Cyp3a25	CCGTTACTTGGCACCATTTT / GTCTTTCATGCTGATGGGCT	390	D. Choudhary et. al. (2003)	
Cyp4a10	TATGTGAAAAACATGGCCGA / TCTTTTCCAGCTCTCCCTCA	376	D. Choudhary et. al. (2003)	
Cyp4a14	GATGTTGACTCCAGCCTTCC / CATTCTGCAGCTGAGACTTCC	398	D. Choudhary et. al. (2003)	
Cyp4b1	TGATGTGCTGAAGCCCTATG / CGCTCCTGAAGCTTTTTCTG	399	D. Choudhary et. al. (2003)	
Cyp4f13	GGCCTTGATGAAGAACAACG / AAACATGTCCAGACGGGAAG	385	D. Choudhary et. al. (2003)	
Cyp4f14	GGTACCTACCCCCAAGGTTT / GCTGTCAAAGCTGAAGACACA	393	D. Choudhary et. al. (2003)	
Ugt1a1	GCTTCTTCCGTACCTTCTGTTG / GCTGCTGAATAACTCCAAGCAT	119	Jialin Xu et. al. (2012)	
Ugt1a2	ATGGACACGGGACTATGTGTG / CATGGGTAACACCAGCACTTTT	105	Jialin Xu et. al. (2012)	
Utg1a5	TGAGAAGGTGCTAGTGTTTCCT / GGGAACGGCATAGACTTTGAA	166	PrimerBank ID : 145699136c1	
Ugt1a6	GTTTCTCTTCCTAGTGCTTTGGG / CCTCGTTCACTGAGATGTTCTAC	117	Jialin Xu et. al. (2012)	
Ugt1a7	CCACTGGTTTACGATGCAGAC / CCTCCTGCGTATGAGAAACTG	146	Jialin Xu et. al. (2012)	
Ugt1a9	TCTCGCTCCCATCAGTAATCTT / TGGTTCCACACTCTCTCCTTG	148	Jialin Xu et. al. (2012)	
Ugt2a1	AGCCTTCTAGGAATGAGTCTTGG / CAAGGACAGTCACATTATGCTCT	124	PrimerBank ID : 16716477a1	
Ugt2a2	TGTGGTAGTTTGGCCTACAGA / TTGTGTACGAAACCGGAATCTC	173	PrimerBank ID : 66571302c1	
Ugt2a3	CGTGTGGCCCTGTGATATGAG / GTGCAGTGGAATACGTTTACTCT	136	Jialin Xu et. al. (2012)	
Ugt2b1	GTGCTGGTGTGGCCTACAG / ATTGCTCGGCCCAATGAGG	129	Jialin Xu et. al. (2012)	
Ugt2b5	ACGAGGCGATCTATCATGGAA / GACCTCCTCCAGTGCATTGAG	155	Jialin Xu et. al. (2012)	
Ugt2b34	TGAAGTGATGGTTCTGAGACCT / GCAGATGTGTTGTCAACCTCATA	60	Jialin Xu et. al. (2012)	


Name	Sequences (Forward / Reverse)	Product Size (base pairs)	References	
Ugt2b35	CCTGCTAAGCCCTTGCCTAAG / AAATTGCGTTGGCCCTTTCTT	124	PrimerBank ID : 27370342a1	
Ugt2b36	CAAGTGGCTTCCCCAAAATGA / ATGGATCGCCTCGTAGAGTCC	94	Jialin Xu et. al. (2012)	
Ugt3a1	AAACGCCCCCTTGTCATATG / CCTTCGCTTCTTGGTGAAATG	115	Jialin Xu et. al. (2012)	
Ugt3a2	CACTCATGGAGGGATGAACAGT / TGGTGAGCGCAAATGACTCTG	170	Jialin Xu et. al. (2012)	
Sult1a1	CAACATGGAGCCCTTGCGTAA / ATGAGCACATCATCAGGCCAG	120	PrimerBank ID : 5420463a1	
Sult1b1	GCACACCAGGTGACATTGTAA / CCGAGGTGATGGAGTTTTCTTC	206	PrimerBank ID : 9845263a1	
Sult1c1	AACATGCAGCCAGAAACCAG / GTCCACAATTTCCTGTGTCCA	159	PrimerBank ID : 9055354a1	
Sult1c2	ATGGCCTTGACCCCAGAAC / TCGAAGGTCTGAATCTGCCTC	107	PrimerBank ID : 34328501a1	
Sult1d1	ATGTCTTCAGGAGGGAGTTAGTG / CATCAGGCCGGGCTTCAAA	102	PrimerBank ID : 7949146a1	
Sult1e1	ATGGAGACTTCTATGCCTGAGT / ACACAACTTCACTAATCCAGGTG	175	PrimerBank ID : 12963515a1	
Sult2a2	TAACTTACCCCAAGTCAGGAACG / ATGGGAAGATGGGAGGTTATGA	187	PrimerBank ID : 6678159a1	
Sult2b1	TCCTGTCGGCATGTACTCAC / CGCACGTTGCTAGTGTTCTC	60	PrimerBank ID : 8567408a1	
Sult3a1	TATTTTGAGGGTCATCGGAACAG / GGTGATGGCATTTTGGCATAGT	107	PrimerBank ID : 10181194a1	
Sult4a1	CCTGGGTGTGTCCTGTGATAA / CACTTCCCCATCTTCTGTTTGT	189	PrimerBank ID : 34328449a1	
Sult5a1	ATGACTGAGCGCATGAACACC / CCACAAGTGACCCTCACAGA	198	PrimerBank ID : 10181192a1	
Mdr1a	CAGCAGTCAGTGTGCTTACAA / ATGGCTCTTTTATCGGCCTCA	205	PrimerBank ID : 6755048a1	
Bcrp	GAACTCCAGAGCCGTTAGGAC / CAGAATAGCATTAAGGCCAGGTT	166	PrimerBank ID : 6752944a1	
Oatp1b3	GGGAACATGCTTCGTGGGATA / GGAGTTATGCGGACACTTCTC	218	PrimerBank ID : 14547899a1	
Oatp2b1	CTCAGGACTCACATCAGGATGC / CTCTTGAGGTAGCCAGAGATCA	118	PrimerBank ID : 29789421a1	
Mate1	TGCGGTTATCAATGTCACAGG / CTACGTGCTTTAAGTTCTGGCT	104	PrimerBank ID : 21594552a1	
Bsep	TCTGACTCAGTGATTCTTCGCA / CCCATAAACATCAGCCAGTTGT	191	PrimerBank ID : 11038656a1	
Mrp2	GTGTGGATTCCCTTGGGCTTT / CACAACGAACACCTGCTTGG	123	PrimerBank ID : 11184219a1	
Mrp3	CTGGGTCCCCTGCATCTAC / GCCGTCTTGAGCCTGGATAAC	111	PrimerBank ID : 29179622a1	
Mrp4	CATCGCGGTAACCGTCCTC / CCGCAGTTTTACTCCGCAG	134	PrimerBank ID : 255683319c3	
Ent1	CAGCCTCAGGACAGGTATAAGG / GTTTGTGAAATACTTGGTTGCGG	108	PrimerBank ID : 12584968a1	
Pept1	AGATGGTCACGAAGGTGATGT / TTCCCATTCATGGTCGTTGCT	109	PrimerBank ID : 16716359a1	

a, b: The result of the RT-PCR for Cyp2b10 was validated by two different Cyp2b10 primer sets, and essentially same result was observed. Fig. 2 is the data using the primer set marked with “a”, and Fig. 3 and Table 1 is the data using the primer set marked with “b”.

References: Primers were designed referring to previous reports and Primer Bank (https://pga.mgh.harvard.edu/primerbank)  (Choudhary  2003;  Wang  and  Seed  2003;  Spandidos  2008;
Spandidos 2010; Xu 2012).
