# Supplementary material for: Daikenchuto (TU‐100) alters murine hepatic and intestinal drug metabolizing enzymes in an in vivo dietary model: effects of gender and withdrawal
Source: Pharmacol Res Perspect. 2017 Oct 3;5(5):e00361. doi: 10.1002/prp2.361 (PMC5625165; doi:10.1002/prp2.361)
Supplement: Supplementary file 2 — Table S2. Effect of TU‐100 on Drug Metabolizing Enzymes/Phase II. Table S3. Effect of TU‐100 on drug transporters. [file PRP2-5-e00361-s002.docx]

**Table 1. Effect of TU-100 on Drug Metabolizing Enzymes/Cytochromes Phase 1**

|  | MALE | | | FEMALE | | |
| --- | --- | --- | --- | --- | --- | --- |
|  | 12 weeks AIN76 with TU-100 | | | | | |
|  | Liver | Jejunum | Prox colon | Liver | Jejunum | Prox Colon |
| Cyp1a1 | 1.22±0.26 | 0.98±0.19 |  | 2.27±0.26** | 1.30±0.27 |  |
| Cyp1a2 | 0.97±0.09 |  |  | 1.29±0.14 |  |  |
| Cyp2a4 | 1.47±0.18 |  |  | 1.38±0.15 |  |  |
| Cyp2b10 | 1.88±0.39 | 1.34±0.13** |  | 6.94±0.97 | 1.94±0.23** |  |
| Cyp2c9 | 1.23±0.10 |  |  | 1.34±0.11* |  |  |
| Cyp2c37 | 1.02±0.07 |  |  | 1.41±0.02* |  |  |
| Cyp2c40 | 0.78±0.04 | 0.96±0.10 | 1.39±0.18 | 0.91±0.08 | 1.07±0.08 | 1.88±0.37* |
| Cyp2d9 | 0.92±0.06 |  | 0.63±0.05 | 1.14±0.09 |  | 0.82±0.07 |
| Cyp2d26 | 1.03±0.09 | 1.04±0.10 | 0.83±0.11 | 1.28±0.08 | 1.24±0.09 | 0.64±0.08 |
| Cyp3a11 | 1.46±0.95 | 1.18±0.09 | 0.37±0.07** | 1.51±0.12** | 1.63±0.20** | 0.85±0.09 |
| Cyp3a13 | 0.95±0.08 | 1.22±0.10 | 1.27±0.14 | 1.19±0.13 | 1.07±0.07 | 1.29±0.14 |
|  |  |  |  |  |  |  |
|  | MALE | | | FEMALE | | |
|  | 24 weeks AIN76 with 1.5% TU-100 | | |  |  |  |
|  | Liver | Jejunum | Prox colon | Liver | Jejunum | Prox Colon |
| Cyp1a1 | 1.94±0.21** | 1.07±0.18 |  | 0.72±0,09 | 1.22±0.29 |  |
| Cyp1a2 | 1.00±0.09 |  |  | 1.10±0.09 |  |  |
| Cyp2a4 | 3.99±0.87** |  |  | 1.06±0.11 |  |  |
| Cyp2b10 | 3.07±0.82** | 1.83±0.26* |  | 5.68±0.80** | 4.55±0.79** |  |
| Cyp2c9 | 0.66±0.07* |  |  | 0.75±0.05* |  |  |
| Cyp2c37 | 1.00±0.12 |  |  | 1.15±0.90 |  |  |
| Cyp2c40 | 0.86±0.08 | 1.14±0.15 | 1.60±0.22 | 0.90±0.12 | 1.56±0.21 | 1.25±0.17 |
| Cyp2d9 | 0.72±0.07* |  | 1.16±0.15 | 0.87±0.07 |  | 0.93±0.13 |
| Cyp2d26 | 0.77±0.09 | 0.93±0.09 | 0.85±0.17 | 1.31±0.18 | 1.60±0.30 | 1.56±0.32 |
| Cyp3a11 | 1.79±0.39 | 1.05±0.14 | 0.32±0.07** | 1.29±0.15 | 1.75±0.25* | 1.37±0.22 |
| Cyp3a13 | 0.80±0.19 | 0.95±0.06 | 0.80±0.08 | 1.57±0.12* | 1.16±0.18 | 1.81±0.30* |
|  |  |  |  |  |  |  |
|  | MALE | | | FEMALE | | |
|  | 12 weeks AIN76 with 1.5% TU-100/12 weeks of AIN76 | | | | | |
|  | Liver | Jejunum | Prox colon | Liver | Jejunum | Prox Colon |
| Cyp1a1 | 0.80±0.12 | 0.84±0.07 |  | 0.70±0.12 | 1.46±0.30 |  |
| Cyp1a2 | 1.32±0.19 |  |  | 0.76±0.09 |  |  |
| Cyp2a4 | 1.16±0.13 |  |  | 1.11±0.16 |  |  |
| Cyp2b10 | 0.66±0.06 | 0.97±0.13 |  | 0.53±0.20 | 1.28±0.16 |  |
| Cyp2c9 | 0.85±0.04 |  |  | 0.83±.011 |  |  |
| Cyp2c37 | 0.99±0.10 |  |  | 1.00±0.16 |  |  |
| Cyp2c40 | 1.10±0.08 | 0.78±0.06 | 1.90±0.40 | 1.06±0.18 | 1.82±0.29* | 1.77±0.32* |
| Cyp2d9 | 0.87±0.07 |  | 1.79±0.19* | 0.95±0.11 |  | 1.27±0.11 |
| Cyp2d26 | 0.82±0.05* | 0.80±0.05 | 0.83±0.23 | 1.51±.015* | 1.50±0.13* | 0.93±.019 |
| Cyp3a11 | 0.97±0.09 | 1.00±0.09 | 0.89±0.20 | 1.08±0.20 | 1.17±0.09 | 0.63±0.13 |
| Cyp3a13 | 0.78±0.11 | 1.06±0.14 | 1.06±0.10 | 1.34±0.20 | 1.01±0.09 | 1.43±0.26 |

**Table 1. Effect of Dietary TU-100 on Cytochrome Gene Expression. Male and female mice were fed AIN76A diet without TU-100 or with 1.5% TU-100 for 12 (middle panels) or 24 weeks (middle panels) or 12 weeks with TU-100 followed by 12 weeks without TU-100 (reversal, bottom panels and compared with expression with diet without TU-100.**

**Table 2. Effect of TU-100 on Drug Metabolizing Enzymes/Phase II**

|  | MALE | | | FEMALE | | |
| --- | --- | --- | --- | --- | --- | --- |
|  | 12 weeks AIN76 with TU-100 | | | | | |
|  | Liver | Jejunum | Prox colon | Liver | Jejunum | Prox Colon |
| Ugt1a1 | 0.94±0.09 | 1.05±0.10 | 0.99±0.09 | 1.28±0.12 | 1.05±0.10 | 0.78±0.07 |
| Ugt1a5 | 0.86±0.07 | 0.71±0.09 | 0.48±0.06 | 1.27±0.16 | 0.70±0.09 | 1.89±0.32** |
| Ugt1a6 | 0.75±0.05* | 0.68±0.06* | 1.19±0.17 | 1.00±0.08 | 1.57±0.24* | 0.92±0.09 |
| Ugt2b1 | 0.59±0.05** |  |  | 0.09±0.09 |  |  |
| Ugt2b5 | 0.72±0.04 | 0.94±0.07 | 1.08±0.13 | 1.72±0.25* | 1.69±0.16* | 0.76±0.07 |
| Ugt2b34 | 0.94±0.08 | 1.09±0.08 | 1.18±0.10 | 0.86±0.07 | 0.94±0.10 | 0.67±0.06** |
| Ugt2b36 | 0.72±0.06 | 1.04±0.11 | 1.23±0.09 | 1.25±0.12 | 1.57±0.16** | 0.79±0.07 |
| Ugt2a3 | 0.93±0.08 | 0.82±0.08 |  | 1.04±0.08 | 1.41±0.12* |  |
| Ugt3a2 | 0.70±0.05 | 0.61±0.11 | 0.26±0.03* | 0.82±0.06 | 1.79±0.19* | 3.61±0.78** |
| Sult1a1 | 0.56±0.05* |  | 1.04±0.06 | 0.92±0.10 |  | 0.91±0.09 |
| Sult1a2 | 0.68±0.04 | 0.76±0.04* | 0.81±0.09 | 1.41±0.16* | 1.08±0.09 | 1.00±0.11 |
|  |  |  |  |  |  |  |
|  | MALE | | | FEMALE | | |
|  | 24 weeks AIN76 with 1.5% TU-100 | | |  |  |  |
|  | Liver | Jejunum | Prox colon | Liver | Jejunum | Prox Colon |
| Ugt1a1 | 1.14±0.19 | 1.22±0.16 | 0.92±0.11 | 0.85±0.07 | 1.76±0.37 | 1.37±0.25 |
| Ugt1a5 | 0.86±0.10 | 1.56±0.20 | 1.16±0.13 | 0.68±0.10 | 1.60±0.32 | 2.58±0.65* |
| Ugt1a6 | 1.12±0.09 | 1.38±0.14 | 1.31±0.15 | 1.02±0.14 | 1.16±0.18 | 0.89±0.12 |
| Ugt2b1 | 0.60±0.08** |  |  | 0.88±0.05 |  |  |
| Ugt2b5 | 1.70±0.28* | 1.40±0.25 | 0.74±0.07 | 0.68±0.04°° | 1.12±0.13 | 1.25±0.16 |
| Ugt2b34 | 1.08±0.11 | 1.37±0.16 | 1.02±0.27 | 1.03±0.10 | 1.04±0.14 | 0.94±0.09 |
| Ugt2b36 | 1.12±0.28 | 0.90±0.08 | 0.85±0.07 | 1.12±0.06 | 1.34±0.22 | 1.52±0.22* |
| Ugt2a3 | 1.06±0.20 | 1.49±0.22 |  | 1.15±0.04 | 1.27±0.23 |  |
| Ugt3a2 | 1.02±0.09 | 0.89±0.09 | 0.63±0.13 | 0.94±0.08 | 0.95±0.25 | 1.22±0.27 |
| Sult1a1 | 0.81±0.23 |  | 0.94±0.17 | 1.17±0.10 |  | 0.96±0.17 |
| Sult1a2 | 1.05±0.22 | 1.94±0.28** | 0.95±0.13 | 1.22±0.15 | 0.76±0.09 | 1.10±0.15 |
|  |  |  |  |  |  |  |
|  | MALE |  |  | FEMALE |  |  |
|  | 24 weeks AIN76 with 1.5% TU-100 | | | | | |
|  | Liver | Jejunum | Prox colon | Liver | Jejunum | Prox Colon |
| Ugt1a1 | 0.77±0.05 | 1.02±0.06 | 1.18±0.28 | 0.94±0.14 | 1.70±0.14* | 0.67±0.08* |
| Ugt1a5 | 0.70±0.06* | 0.88±0.15 | 1.33±0.21 | 0.79±0.13 | 1.21±0.14 | 1.84±0.40 |
| Ugt1a6 | 1.09±0.06 | 0.84±0.04 | 1.12±0.27 | 1.04±0.13 | 1.02±0.12 | 0.68±0.15 |
| Ugt2b1 | 1.08±0.05 |  |  | 1.18±0.14 |  |  |
| Ugt2b5 | 1.62±0.57 | 1.13±0.15 | 0.89±0.22 | 0.72±0.14* | 1.89±0.35* | 1.14±0.22 |
| Ugt2b34 | 0.83±0.06* | 1.20±0.13 | 0.99±0.18 | 1.04±0.16 | 1.11±0.15* | 1.17±0.15 |
| Ugt2b36 | 1.08±0.15 | 0.84±0.10 | 0.94±0.13 | 1.14±0.07 | 1.48±0.23 | 1.13±0.16 |
| Ugt2a3 | 1.03±0.06 | 1.21±0.10 |  | 1.28±0.15 | 1.81±0.28* |  |
| Ugt3a2 | 1.17±0.20 | 1.15±0.18 | 1.15±0.15 | 1.19±0.16 | 1.02±0.16 | 1.19±0.12 |
| Sult1a1 | 0.66±0.05 |  | 0.82±0.10 | 1.27±0.15 |  | 1.02±0.13 |
| Sult1a2 | 1.04±0.19 | 1.58±0.14* | 1.83±0.19* | 1.11±0.12 | 1.08±0.09 | 1.00±0.08 |

**Table 2.** **Effect of Dietary TU-100 on Phase II Drug Metabolizing enzymes. Male and female mice were fed AIN76A diet without TU-100 or with 1.5% TU-100 for 12 (middle panels) or 24 weeks (middle panels) or 12 weeks with TU-100 followed by 12 weeks without TU-100 (reversal, bottom panels and compared with expression with diet without TU-100.**

**Table 3. Effect of TU-100 on Drug Transporters**

|  | MALE | | | | FEMALE | | | |
| --- | --- | --- | --- | --- | --- | --- | --- | --- |
|  | 12 weeks AIN76 with TU-100 | | | | | | | |
|  | Liver | Jejunum | | Prox colon | Liver | Jejunum | Prox Colon | |
| Mdr1a | 1.20±0.11 | 1.84±0.24** | | 1.48±0.11** | 2.11±0.21** | 1.71±0.24* | 1.15±0.07 | |
| Brcp | 0.86±0.04 | 0.81±0.07 | | 0.99±0.10 | 1.56±0.10** | 1.04±0.09 | 1.19±0.07 | |
| Oatp1b3 | 0.66±0.04** |  | |  | 1.13±0.11 |  |  | |
| Oatp2b1 | 0.74±0.06* | 0.73±0.07 | | 0.76±0.09 | 0.98±0.11 | 0.89±0.08 | 0.86±0.09 | |
| Mate1 | 0.77±0.05* |  | |  | 1.23±0.12 |  |  | |
| Bsep | 0.60±0.06** |  | |  | 1.20±0.09 |  |  | |
| Mrp2 | 0.54±0.04** | 1.62±0.19** | |  | 1.18±0.10 | 1.38±0.11 |  | |
| Mrp3 | 1.43±0.14 | 1.03±0.06 | | 1.05±0.15 | 2.02±0.16** | 1.30±0.10 | 0.95±0.06 | |
| Ent1 | 0.69±0.04** |  | | 0.80±0.07 | 1.20±0.10 |  | 0.76±0.08* | |
| Pept1 |  | 0.79±0.09 | | 0.72±0.10 |  | 1.26±0.12 | 1.55±0.25 | |
|  |  |  | |  |  |  |  | |
|  | MALE | | | | FEMALE | | | |
|  | 24 weeks AIN76 with 1.5% TU-100 | | | |  |  | |  |
|  | Liver | | Jejunum | Prox colon | Liver | Jejunum | | Prox Colon |
| Mdr1a | 3.83±0.59** | | 4.41±0.82** | 2.25±0.40** | 1.22±0.15 | 1.38±0.22 | | 2.00±0.36* |
| Brcp | 1.36±0.09** | | 0.93±0.13 | 0.88±0.09 | 1.13±0.08 | 1.20±0.20 | | 1.54±0.19* |
| Oatp1b3 | 1.03±0.14 | |  |  | 0.89±0.09 |  | |  |
| Oatp2b1 | 1.14±0.07 | | 1.32±0.19 | 0.83±0.11 | 0.87±0.13 | 1.27±0.17 | | 1.15±0.17 |
| Mate1 | 0.74±0.17 | |  |  | 0.95±0.03 |  | |  |
| Bsep | 0.75±0.11 | |  |  | 0.81±0.06 |  | |  |
| Mrp2 | 0.92±0.11 | | 3.00±0.36** |  | 1.02±0.02 | 0.82±0.08 | |  |
| Mrp3 | 2.12±0.40* | | 2.77±0.32** | 1.10±0.11 | 1.11±0.09 | 0.98±0.16 | | 1.31±0.17 |
| Ent1 | 0.69±0.04* | |  | 0.80±0.11 | 0.73±0.08 |  | | 1.31±0.18 |
| Pept1 |  | | 2.78±0.49** | 1.32±0.15 |  | 1.26±0.32 | | 0.96±0.10 |
|  |  | |  |  |  |  | |  |
|  | MALE | | | | FEMALE | | | |
|  | 12 weeks AIN76 with 1.5% TU-100/12 weeks of AIN76 | | | | | | | |
|  | Liver | | Jejunum | Prox colon | Liver | Jejunum | | Prox Colon |
| Mdr1a | 0.83±0.07 | | 1.16±0.10 | 1.12±0.26 | 0.90±0.13 | 3.11±0.75** | | 0.90±0.16 |
| Brcp | 1.09±0.16 | | 0.91±0.05 | 1.10±0.13 | 0.77±0.06 | 1.14±0.14 | | 1.31±0.23 |
| Oatp1b3 | 0.87±0.08 | |  |  | 1.17±0.18 |  | |  |
| Oatp2b1 | 0.90±0.10 | | 0.89±0.08 | 0.84±0.06 | 1.02±0.09 | 1.02±0.05 | | 1.10±0.12 |
| Mate1 | 0.86±0.11 | |  |  | 0.91±0.14 |  | |  |
| Bsep | 0.79±0.08 | |  |  | 1.29±0.14 |  | |  |
| Mrp2 | 0.68±0.08* | | 1.05±0.09 |  | 1.15±0.08 | 1.62±0.15** | |  |
| Mrp3 | 0.79±0.08 | | 1.42±0.13* | 1.15±0.23 | 1.06±0.13 | 1.10±0.17 | | 1.32±0.18 |
| Ent1 | 0.88±0.07 | |  | 0.93±0.08 | 0.82±0.13 |  | | 1.35±0.18 |
| Pept1 |  | | 1.14±0.17 | 2.18±0.53 |  | 1.88±0.12** | | 2.73±0.71* |

**Table 3.**  **Effect of Dietary TU-100 on Drug Transporters. Male and female mice were fed AIN76A diet without TU-100 or with 1.5% TU-100 for 12 (middle panels) or 24 weeks (middle panels) or 12 weeks with TU-100 followed by 12 weeks without TU-100 (reversal, bottom panels and compared with expression with diet without TU-100.**
